# Synchrotron Characterization of Hexagonal and Cubic Lipidic Phases Loaded with Azolate/Phosphane Gold(I) Compounds: A New Approach to the Uploading of Gold(I)-Based Drugs

**DOI:** 10.3390/nano10091851

**Published:** 2020-09-16

**Authors:** Paola Astolfi, Michela Pisani, Elisabetta Giorgini, Barbara Rossi, Alessandro Damin, Francesco Vita, Oriano Francescangeli, Lorenzo Luciani, Rossana Galassi

**Affiliations:** 1 Dipartimento SIMAU, Università Politecnica delle Marche, Via Brecce Bianche, I-60131 Ancona, Italy; p.astolfi@univpm.it (P.A.); f.vita@univpm.it (F.V.); o.francescangeli@univpm.it (O.F.); 2 Dipartimento DiSVA, Università Politecnica delle Marche, Via Brecce Bianche, I-60131 Ancona, Italy; e.giorgini@univpm.it; 3 Elettra-Sincrotrone Trieste S.C.p.A., S.S. 14-km 163.5, Basovizza, I-34149 Trieste, Italy; barbara.rossi@elettra.eu; 4 Department of Chemistry, NIS Centre and INSTM Reference Centre University of Turin, Via G. Quarello 15, I-10135 Turin, Italy; alessandro.damin@unito.it; 5 Scuola di Scienze e Tecnologie, Divisione Chimica, Università di Camerino, I-62032 Via Sant’Agostino 1, Italy; lorenzo.luciani@unicam.it (L.L.); rossana.galassi@unicam.it (R.G.)

**Keywords:** lyotropic cubic and hexagonal mesophases, gold compounds, drug delivery, phytantriol, monoolein, DOPE

## Abstract

Gold(I) phosphane compounds have recently attracted a renewed interest as potential new protagonists in cancer therapy. A class of phosphane gold(I) complexes containing azolate ligands has been successfully tested against several cancer cell lines and, in particular, against basal-like breast (BLB) cancer, a form characterized by strongly severe diagnosis and short life lapse after classic chemotherapy. Even though the anticancer activity of gold(I) phosphane compounds is thoroughly ascertained, no study has been devoted to the possibility of their delivery in nanovectors. Herein, nonlamellar lyotropic liquid crystalline lipid nanosystems, a promising class of smart materials, have been used to encapsulate gold(I) azolate/phosphane complexes. In particular, ((triphenylphosphine)-gold(I)-(4,5-dichloroimidazolyl-1H-1yl)) (C-I) and ((triphenylphosphine)-gold(I)-(4,5-dicyanoimidazolyl-1H-1yl)) (C-II) have been encapsulated in three different lipid matrices: monoolein (GMO), phytantriol (PHYT) and dioleoyl-phosphatidylethanolamine (DOPE). An integrated experimental approach involving X-ray diffraction and UV resonant Raman (UVRR) spectroscopy, based on synchrotron light and attenuated total reflectance Fourier transform infrared (ATR-FTIR) spectroscopy, has been employed to establish the effects of drug encapsulation on the structure and phase behavior of the host mesophases. The results indicate that gold(I) complexes C-I and C-II are successfully encapsulated in the three lipid matrices as evidenced by the drug-induced phase transitions or by the changes in the mesophase lattice parameters observed in X-ray diffraction experiments and by the spectral changes occurring in UV resonant Raman spectra upon loading the lipid matrices with C-I and C-II.

## 1. Introduction

A continuous research effort is in progress for the production of efficient drug nanocarriers for anticancer therapy having formulations with a higher degree of specificity and reduced adverse effects. In particular, lyotropic liquid crystalline phases represent interesting systems since they assemble in a multitude of architectures, characterized by the coexistence of separate hydrophobic and hydrophilic domains. The most known and widely employed for drug delivery are liposomes. [1,2,3,4]. More recently, cubosome and hexosome systems, in which the lipid bilayers are arranged in periodic two and three dimensional lattice structures, represent attractive smart delivery matrices for drugs and/or diagnostic agents with different water affinity [5,6,7,8,9,10,11,12,13]. The latter structures have received increasing attention due to their properties, such as size, structure, and stability; ability to be tuned by their internal composition, polymer concentration, and processing conditions; and their ability to accommodate relatively large loads (drug or proteins). Moreover, the stiffness of these phases can lead to a slower diffusion of the solubilized cargo and hence to a long-term release. Within this frame, we are currently developing bio-systems based on hexagonal and cubic phases dispersed in a continuous aqueous medium, which could find a potential application as anticancer drug delivery vectors. As an example, phytantriol cubic phases have been successfully used to encapsulate the commercial antineoplastic 5-fluororuracil [14,15]. Hence, to explore the full potential of these systems, our attention has been focused on promising anticancer drugs based on gold(I) phosphane complexes.

These latter compounds have shown anti-neoplastic effects on many cancer cells panels and unique in vitro ability to induce a dose-dependent inhibition of cell proliferation in both BLBC (breast cancer basal like) murine A17 and human MDA-MB-231 cells, also confirmed by in vivo tests [16,17]. BLBC is a particularly aggressive subtype of breast cancer associated with very short relapse-free survival, and finding an effective therapy to prolong this time is still a challenge. In this regard, gold phosphane compounds could be valid candidates to be tested in the case of intolerance and resistance to classic chemotherapy agents [17], even if obstacles to their use can be represented by the lack of a univocal mechanism of action and, even more so, by the lability of gold phosphane compounds in the presence of cysteine residues of albumins [18,19].

**Figure 1:**
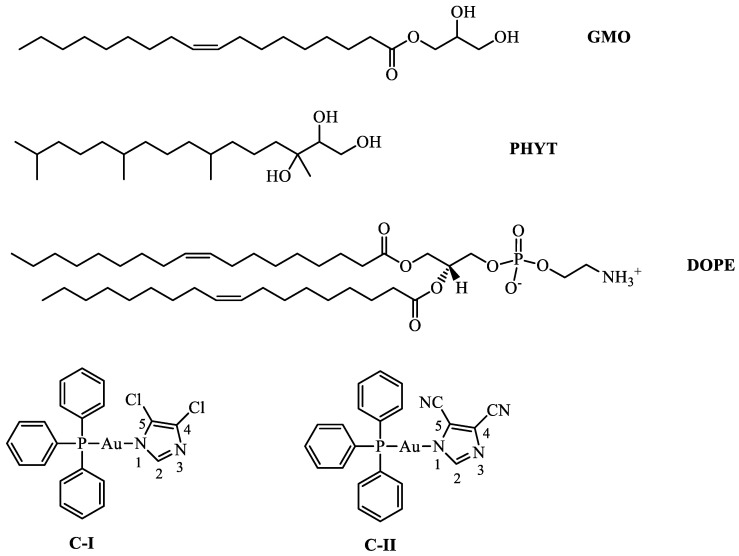
Chemical structures of glyceryl monooleate (GMO), phytantriol (PHYT), 1,2-dioleoyl-sn-glycero-3-phosphoethanolamine (DOPE), ((triphenylphosphine)-gold(I)-(4,5-dichloroimidazolyl-1H-1yl) (C-I) and ((triphenylphosphine)-gold(I)-(4,5-dicyanoimidazolyl-1H-1yl) (C-II).

Many studies on gold compounds focused on the detection of specific molecular targets [20,21], whereas no data have been reported on a possible encapsulation of these compounds, which should prevent the exchange reactions usually occurring with the cysteine groups of serum albumin and which lead to systemic toxicity with homeostatic effects [22] and on their use in drug delivery to improve their stability under physiological conditions [23]. In this study, we attempted a pioneer experimental approach on the encapsulation of ((triphenylphosphine)-gold(I)-(4,5-dichloroimidazolyl-1H-1yl)) (C-I) and ((triphenylphosphine)-gold(I)-(4,5-dicyanoimidazolyl-1H-1yl)) (C-II) in different lipid matrices such as glyceryl monooleate or monoolein (GMO), phytantriol (PHYT) and dioleoyl-phosphatidylethanolamine (DOPE) (Figure 1). A multitask characterization involving small-angle X-ray diffraction (SAXS), UV resonant Raman (UVRR) and attenuated total reflectance Fourier transform infrared (ATR-FTIR) spectroscopy was employed to establish the effects of gold compounds encapsulation on the structure and phase behavior of the mesophases. The SAXS technique is a unique tool able to achieve information on the structural modifications of lipid phases induced by encapsulation of the drug. The vibrational spectroscopic techniques, such as IR and UV resonance Raman scattering, are extremely sensitive to the intra- and inter-molecular interactions, and, therefore, they are very useful for providing insights on the possible molecular arrangements of specific chemical groups of the compounds inside the hexagonal and cubic phases. The interpretation of the vibrational spectroscopy results is supported by the assignment of the main vibrational signals observed in the experimental spectra by theoretical calculations.

## 2. Materials and Methods

*Sample Preparation.* Glyceryl monooleate (GMO) was purchased from Merck (Milan, Italy), phytantriol (PHYT) was purchased from TCI Europe and 1,2-dioleoyl-sn-glycero-3-phosphoethanolamine (DOPE) from Avanti Polar Lipids. (4,5-dicloro-1H-imidazolate-1-yl)-(triphenylphosphane)-gold(I) (C-I) and (4,5-dicyano-1H-imidazolate-1-yl)-(triphenylphosphane)-gold(I) (C-II) were synthesized [24] as previously described; 4,5-dichloro-imidazole and the 4,5-dicyano-imidazole were N–H deprotonated with KOH in THF or with solid K_2_CO_3_ in H_2_O/CH_3_OH solution, respectively, and reacted with PPh_3_AuBF_4_ in THF solution in a 1:1 mole ratio. The crude reaction mixtures were washed with water and the organic phases taken to dryness. Compounds C-I and C-II were crystallized with CH_2_Cl_2_/diethyl ether.

LogP values for C-I and C-II, +0.87 and +0.97, respectively, were experimentally determined by the shake-flask method [25]. C-I or C-II were dissolved in 1-octanol, shaken with an equal volume of water, and left until separation of two phases. Before the usage, both octanol and water were saturated with the respective co-solvent and left to equilibrate for days. Gold compound UV absorbance (λ = 276 nm for C-I and λ = 235 nm for C-II) was measured, and the concentrations were determined by using the corresponding calibration curves for each compound. LogP values for C-I and C-II are given by the logarithm of the ratio between the molar concentration of the sample in the two phases. Pluronic F127, chloroform and methanol were obtained from Merck (Milan, Italy). All chemicals used in this study were of analytical grade and were used without further purification.

Blank bulk Lyotropic Liquid Crystalline (LLC) phases were prepared by co-dissolving the lipid (20 mg) and Pluronic F127 (10% w/w of lipid) in chloroform. After evaporation of the solvent under a stream of nitrogen and further drying under vacuum for 2 h, water (200 μL) was added, and the mixture was equilibrated at room temperature for 24 h.

Bulk LLC phases loaded gold(I) compound were prepared using the same procedure described for the blank bulk LLC phases, with the difference that 1 mL of a CHCl_3_ solution of the gold(I) compound (2 mg of gold(I) compound in 1 mL of CHCl_3_) was added to the lipid/F127 chloroform mixture before evaporation of the solvent. The retention of the gold(I) compound integrity during the preparation of the samples was verified by ^31^P and ^1^H NMR spectroscopy (see Supplementary Materials).

*NMR Experiment.* The ^1^H and ^31^P NMR spectra were recorded on an Oxford-400 Varian spectrometer (400.4 MHz for ^1^H and 162.1 MHz for ^31^P); the chemical shifts, in ppm, are relative to internal Me_4_Si for ^1^H NMR spectra and to an 85% H_3_PO_4_ standard for ^31^P NMR spectra. Then, 20 mg of GMO was dissolved in 1 mL of CDCl_3_ directly in the NMR tube, followed by the addition of about 2 mg of F127. To this solution, 2 mg of C-I or C-II was added and the ^1^H and ^31^P NMR spectra were acquired. Afterwards, the colorless solutions were evaporated to dryness. The oily residues were treated each with 0.7 mL of deuterated water. To help the homogenization of the suspensions, 10 min long sonication was performed with a bath sonicator (Ney Ultrasonik, 85W). ^1^H spectra in CDCl_3_ were acquired and are reported in the Supplementary Information Section; they showed resolved signals for the GMO/F127and for the compounds C-I or C-II in quite good agreement with the relative concentration of the species in solution (GMO/gold compound 10:1, see Supplementary Materials Figures S1–S5). Addition of D_2_O induced the broadening of the signals (Figures S6 and S7). ^31^P NMR spectra of C-I and C-II in CDCl_3_ are reported in Figures S8 and S9 and are compared with the ^31^P NMR spectra in CDCl_3_ of the mixtures where the peak at around 34 ppm was attributed to the whole azolate/phosphane C-I or C-II compounds (Figures S10 and S11).

*Small-Angle X-ray Scattering.* SAXS measurements were carried out at the high brilliance beamline ID02 at the European Synchrotron Radiation Facility (Grenoble, France). Two-dimensional (2D) diffraction patterns were recorded by a Rayonix MX-170HS detector using an incident beam energy of 12.5 keV (corresponding to the wavelength λ = 0.995 Å) and a sample-to-detector distance of 1.0 m. The investigated *q* range was comprised between 0.3 and 6 nm^−1^ (q=4πsinθ/λ, where 2*θ* is the scattering angle). Measurements were performed in 1 mm diameter glass capillaries at 25 °C and with a maximum exposure time of 1 s/frame to avoid radiation damage. The collected 2D diffraction spectra were angularly integrated to obtain 1D intensity versus *q* curves. The cubic lattice parameter *a* was calculated through the linear fit of *q_hkl_* data vs. $\sqrt{h^{2}+k^{2}+l^{2}}$, whereas the corresponding lattice parameter for the hexagonal phase was obtained from the linear fit of *q_hk_* data vs. $\sqrt{h^{2}+k^{2}+hk}$.

*UV Resonance Raman.* UVRR spectra were collected using the synchrotron-based UVRR set-up available at the BL10.2-IUVS beamline of Elettra Sincrotrone Trieste (Italy) [26]. The exciting wavelength was set at 228 nm by regulating the undulator gap and using a Czerny-Turner monochromator (Acton SP2750, Princeton Instruments, Acton, MA, USA) equipped with a holographic grating with 1800 groves/mm for monochromatizing the incoming SR. The wavelength resolution at 228 nm provided by the monochromator is ≈0.014 nm, which corresponds to a width of ≈2.7 cm^−1^. The final radiation power on the samples was kept at about 10 μW. UVRR spectra of drugs pristine and loaded in the matrices were obtained in a back-scattered geometry by using a triple-stage spectrometer (Trivista, Princeton Instruments, Acton, MA, USA) with a spectral resolution of 2.3 cm^−1^/pixel. Calibration of the spectrometer was standardized using cyclohexane. Any possible photo-damage effect due to a prolonged exposure of the sample to UV radiation was avoided by continuously spinning the sample cell during the measurements. The UVRR spectra reported in the following are the results of the average of four sets of measurements performed on the same sample. Measurements were also carried out on independent samples, and the same spectra were recorded.

The Raman spectra of C-I and C-II were acquired in the 3500–500 cm^−1^ range by using a Horiba HR 320 instrument with a green laser with fundamental wavelength at 532 nm. The single acquisition time was set to 10 s, and the total number of accumulations was set specifically for each sample until a satisfactory signal/noise ratio was reached.

*Attenuated Total Reflectance Fourier Transform Infrared Spectroscopy.* ATR-FTIR measurements were carried out at the infrared SISSI beamline, Elettra Sincrotrone Trieste (Trieste, Italy), by using the MIRacle Single Reflection ATR box (PIKE technologies) with diamond crystal, equipped with a Vertex 70 interferometer (Bruker Optics GmbH) and a deuterated triglycine sulfate (DTGS) detector. IR analysis was performed on GMO, PHYT and DOPE matrices, and C-I and C-II compounds pristine and loaded in the three matrices. Samples were deposited onto the diamond crystal and were maintained under a continuous stream of nitrogen during the measurement. The analysis of each sample took about 2 min, during which the ATR-FTIR spectra were continuously acquired every 5 s and at room temperature. Each spectrum was acquired in the spectral range 5000–550 cm^−1^, and it was the averaged result of 128 scans (spectral resolution 4 cm^−1^). Before each sample acquisition, the background spectrum was collected on the clean diamond crystal under the same conditions. Raw spectra were corrected for carbon dioxide and water vapor and then vector normalized in the entire spectral range of acquisition using specific OPUS 7.5 routines (Bruker Optics GmbH) [15].

*Theoretical Calculations.* Calculation were performed by adopting the Gaussian 09 software [27] and the pure exchange-correlation PBE [28] DFT-based functional, including the D2 empirical dispersive term [29]. H, C, O, P and Cl atoms were described by a 6-31G(2d,p) standard Pople Gaussian basis set; Au atom were described by Lanl2DZ effective core pseudo-potential and its related basis set. The optimized (no symmetry constraints imposed) C-I and C-II molecules were then used for Raman and IR spectrum calculation.

## 3. Results and Discussion

The structural investigation was carried out by SAXS, UV-Raman and ATR-FTIR measurements to study the influence of the gold(I) compounds on the three different lipid matrices GMO, PHYT and DOPE, and to explore the specific interactions of the gold(I) compounds with the carriers.

### 3.1. SAXS

**Figure 2:**
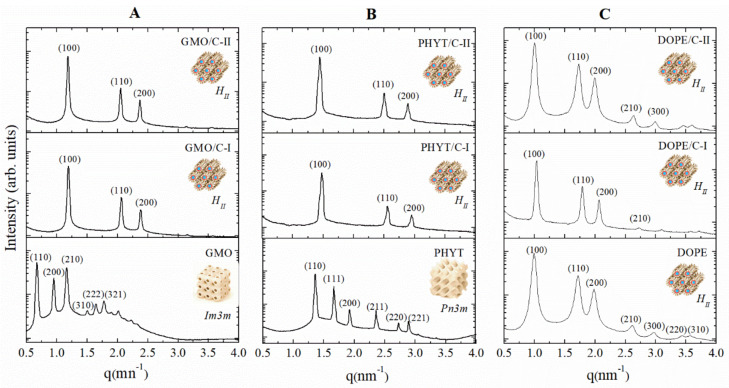
Small-angle X-ray scattering (SAXS) synchrotron profiles for (**A**) GMO, (**B**) PHYT and (**C**) DOPE empty and loaded with C-I and C-II compounds.

SAXS measurements were performed on both empty and loaded GMO, PHYT and DOPE gel phases in excess of water; F127 (10% *w*/*w*) was incorporated in all systems to match the composition of the colloidal dispersions that will be prepared for further studies. The aim of SAXS investigation was to assess if gold compounds arrange in the lipid matrices, each one characterized by a different phase symmetry, by studying the structural changes induced by drug loading. Before encapsulation, the host lyotropic mesophases, GMO, PHYT, and DOPE exhibited *Im3m, Pn3m* and *H_II_* space groups, as indicated by SAXS spectra reported in Figure 2 and in agreement with the literature [30,31,32]. Of the three systems, only GMO was influenced by the addition of the steric stabilizer F127, which induced a phase transition from a *Pn3m* to an *Im3m* bi-continuous cubic phase. This change in the space group is most likely due to a deeper insertion of the hydrophobic polypropylene block (PPO) of F127 into the bilayer of GMO phase [33].

The mesomorphic behavior observed for these systems could be explained by considering their critical packing parameter (*CPP*) that provides a useful measure of the aggregation topology.

*CCP* is defined as: CCP=Va0l where *V* is the hydrophobic chains volume, *a_o_* is the cross-sectional area of hydrophilic headgroup and *l* is the hydrocarbon chain length [34]. Starting from the *CPP* value, amphiphiles can be described according to their “geometrical shape” which, in turn, influences the phase they form. For amphiphilic molecules with *CPP* > 1, inverse-type mesophases are expected (type II), whereas for a *CPP* = 1 and *CPP* < 1, lamellar and normal curved (type I) phases are formed, respectively. In our case, GMO, PHYT and DOPE exhibit a packing parameter larger than 1, thus resulting in reverse self-assembled structures with increasingly negative interfacial curvature, namely the *Im3m*, *Pn3m* and *H_II_* reverse phases. In particular, DOPE exhibits the structure with the most negative interfacial curvature, i.e., the *H_II_* phase. This propensity is likely due to additional inter-bilayer interactions between the charged nitrogen (NH_3_^+^) and phosphate groups (PO_4_^−^) [34]. In this way the effective headgroups cross area is minimized, while the hydrocarbon chains’ volume is increased.

The SAXS patterns of the lipid matrices unloaded and loaded with gold compounds are reported in Figure 2 together with the identification of the different crystallographic symmetries, while the corresponding structural parameters are summarized in Table 1. The water channel radii were estimated using the relation

$$r_{w}=\left[{\left(-\sigma /2\pi \chi \right)}^{1/2}a\right]-l$$

where *l* is the lipid length [15,31,35] (ca. 1.4 nm for PHYT), *a* is the unit cell and σ and χ are topological constants, characteristic of a given cubic phase (for *Pn3m* structure σ = 1.919 and χ = −2, for *Im3m* structure σ = 2.345 and χ = −4). For *H_II_* phase, the radius is given by: $r_{w}=\left(a-d_{l}\right)/2$, where $d_{l}$ is the lipid bilayer thickness along the line connecting the cylinder axes.

The data clearly show that encapsulation of both C-I and C-II compounds strongly affects the structural parameters of all lipid matrices.

However, while loaded GMO and PHYT matrices undergo a phase transition from a cubic to a hexagonal phase, no transition is observed in loaded DOPE. In particular, loaded GMO changes from a cubic *Im3m* symmetry with *a* = 13.24 nm to an inverse hexagonal phase with much reduced lattice parameters, 6.09 nm for GMO/C-I and 6.13 nm for GMO/C-II. A similar phase transition was observed upon addition of C-I and C-II to PHYT: in this case, the system symmetry changes from *Pn3m* with *a* = 6.54 nm to *H_II_* with a unit cell of 4.90 nm for PHYT/C-I and 5.01 nm for PHYT/C-II. Conversely, encapsulation of the gold compounds in the DOPE matrix stabilizes the hexagonal phase, without inducing any phase transition but simply reducing the hexagonal spacing from 7.39 nm for the unloaded DOPE structure to 7.02 nm for DOPE/C-I and 7.25 nm for DOPE/C-II.

Gold compounds C-I and C-II consist of a propeller-shaped hydrophobic PPh_3_ moiety and a more polar imidazole head both bonded to the gold(I) atom in a linear geometry. However, as indicated by their experimental partition coefficients log*P* (+0.87 for C-I and +0.97 for C-II), they are slightly hydrophobic molecules [24], hence preferring to be located in the hydrophobic region of the host matrix bilayers. This insertion causes an increase in the volume of the hydrophobic moiety and induces the transition towards a more negatively curved mesophase, as observed with loaded GMO and PHYT systems. This result is consistent with a previous study, where a transition from a cubic *Im3m* to a reversed hexagonal phase was induced in GMO by encapsulation of a bioactive hydrophobic molecule [36]. When the unloaded system was already in a hexagonal phase, the insertion of C-I and C-II gold compounds results in a more negative curvature of the bilayers and, consequently, in a reduction of both the unit cell parameter and the water channel diameter.

### 3.2. UVRR

**Figure 3:**
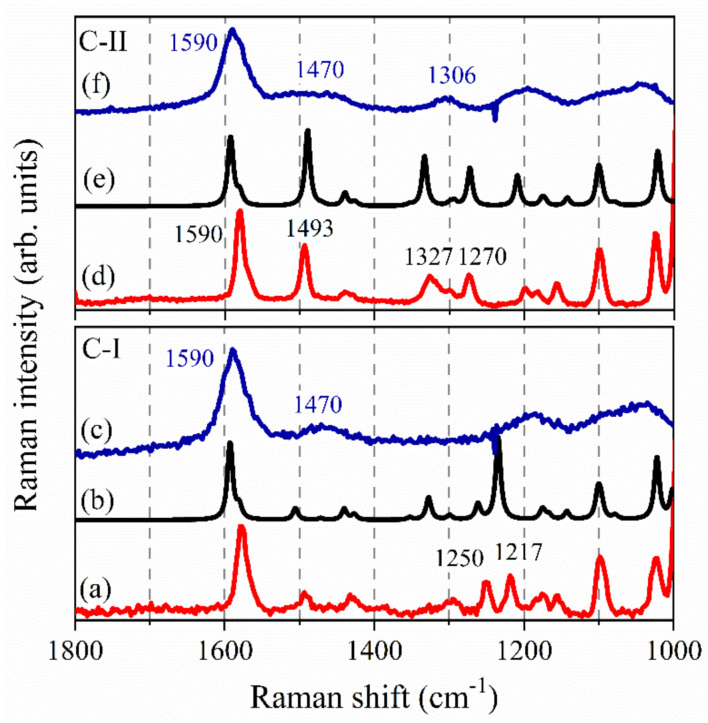
Raman spectra acquired on the pristine drugs C-I (**a**) and C-II (**d**) and theoretical Raman activity computed for the molecules of C-I (**b**) and C-II (**e**) in the spectral region 1100–1800 cm^−1^; UV resonant Raman (UVRR) spectra acquired on C-I (**c**) and C-II (**f**) dissolved in methanol.

Figure 3 shows the comparison among the Raman spectra acquired on the microcrystalline pristine compounds C-I and C-II, the theoretical Raman activity computed for C-I and C-II in the spectral region 1100–1800 cm^−1^ and the experimental UVRR spectra obtained for C-I and C-II dissolved in methanol. Raman spectra of the two compounds are quite similar, especially in the spectral range 1500–1700 cm^−1^ and, by comparing these spectra with the computed ones, an overall match of the number of peaks and of their energy was found. As expected, the UVRR experimental profiles for the methanol solution of the two molecules show marked differences both in the shape of the peaks and in their energy. As common feature to C-I and C-II, the intense Raman peak at ≈1590 cm^−1^ (Figure 3a,d) can reasonably be associated with the corresponding calculated Raman frequencies at ≈1593.7, 1592.5, 1591.8, 1581.1, 1580.3 and 1579.8 cm^−1^. All these vibrations are modes localized on each of the three phenyl rings present in the chemical structure of C-I and C-II, and they are attributed to the deformation modes of the phenyl rings of the AuPPh_3_ moiety. The visible Raman spectrum of C-II clearly shows a signal centered at ≈1493 cm^−1^ that can be recognized also in the profile of C-I, although with a much lower intensity. The comparison between the experimental and calculated Raman frequencies for C-I and C-II suggests that these experimental peaks at ≈1493 cm^−1^ can be attributed to the C4=C5 stretching modes of the imidazolyl ring for both the two molecules. In the visible Raman spectrum of C-II (Figure 3d), two other signals at ≈1327 cm^−1^ and ≈1270 cm^−1^ are visible and are reproduced also in the theoretical Raman spectrum computed for this molecule (falling now at 1333 and 1273 cm^−1^, respectively). From calculations, they can be tentatively assigned to “out of” and “in” phase combination of stretching modes for the C4=N3 and C5=N1 bonds of C-II. As it can be observed from Figure 3a, these same vibrations appear practically Raman inactive in the spectrum of C-I. Conversely, in the visible spectrum of C-I, two Raman signals at ≈1250 cm^−1^ and ≈1217 cm^−1^ are observed, reasonably associated with the calculated Raman frequencies at ≈1234.4 and 1261.5 cm^−1^. These modes correspond to deformation vibrations involving imidazole ring.

As previously mentioned, the experimental UVRR Raman spectra of the two gold compounds appear quite different the Raman ones. This is probably to be ascribed to the resonance effect that causes a selective enhancement of specific chromophores whose electronic transitions are overlapped with the UVRR excitation wavelength at 228 nm. Hence, the use of 228 nm UVRR technique allows one to highlight the phenyl groups’ responses at the expense of other components of the analyte, whose Raman effect may be diminished in intensity or even suppressed [37]. In fact, in our case, the UVRR spectra are dominated by the signals arising from the phenyl ring moieties, while the imidazolyl deformation modes appear very low in intensity. However, only in the case of C-II (Figure 3f) does the experimental UVRR spectrum exhibit an additional Raman component at ≈1306 cm^−1^ that can be reasonably attributed to deformation modes involving the imidazole ring of C-II.

Additionally, the vibrational signals in the UVRR spectra of both C-I and C-II were all broadened compared to the corresponding ones in Raman profiles, and this is likely related to the solvation in methanol of the two molecules and the loosening of the symmetry relationships. Both the UVRR spectra of C-I and C-II molecules exhibit the common spectral feature at ≈1590 cm^−1^ that is attributed to the combination of phenyl ring vibrations. Similarly, the large bump centered in both the spectra of Figure 3c,f at ≈1470 cm^−1^ is probably to be associated with stretching modes of the imidazolyl ring for both the C-I and C-II molecules.

**Figure 4:**
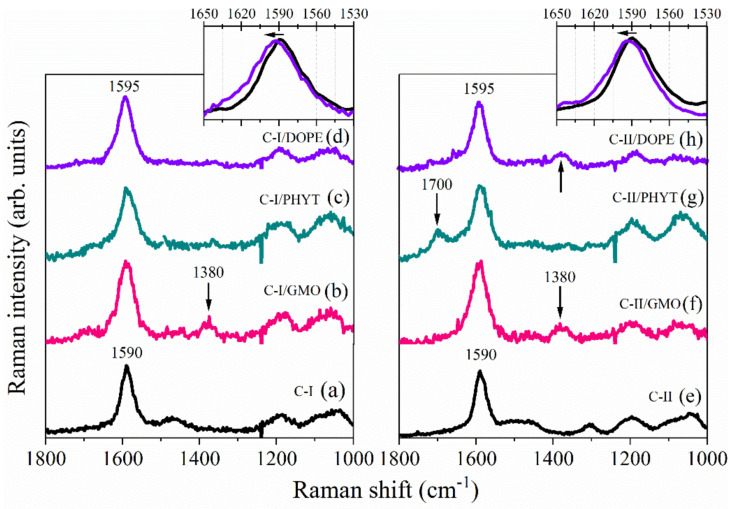
UVRR spectra acquired on pristine drug C-I (**a**) and on the complex formed by C-I with (**b**) GMO, (**c**) PHYT and (**d**) DOPE; on pristine drug C-II (**e**) and on the complex formed by C-II with (**f**) GMO, (**g**) PHYT and (**h**) DOPE. The spectra (**b**–**d**,**f**–**h**) have been subtracted from the lipid matrices signals. Insets: enlargement of the wavenumber region 1650–1530 cm^−1^ for the spectra of C-I and C-I complex with DOPE (left) and spectra of C-II and C-II complex with DOPE (right).

Figure 4a–d point out the spectral changes occurring in the Raman modes of C-I after the loading in the three considered matrices, GMO, PHYT and DOPE. Raman signals coming from the matrices were clearly recognized in the spectra of the complexes and suitably subtracted from the total experimental profile. It was carefully checked that this data handling did not affect the results, but it allowed us to better visualize the modifications occurring on the Raman signal of the molecules after loading.

By looking at the spectral profiles reported in Figure 4a–d, the common behavior of all three complexes in which the loading of C-I in the matrices induces a further broadening of the intense signal at 1590 cm^−1^ was noteworthy. This finding could be correlated to a different environment experienced by one or all the three phenyl rings of C-I when the gold compound is loaded in the matrices. This condition should induce in turn a slight shift of about 5 cm^−1^ in the Raman wavenumber of the phenyl ring deformation modes associated with the Raman band at 1590 cm^−1^. This effect appears particularly evident in the spectrum of C-I complexed with DOPE (Figure 4d) where the blue-shift of the Raman signal, found at 1590 cm^−1^ in the spectrum of the pristine compound, is clearly observed, in agreement with previous results obtained with other systems [38]. This experimental finding is consistent with the establishment of dispersion forces between the phenyl portion of the drug and the lipid phase in the complexes. Figure 4b points out also the rising of a Raman component at ≈1380 cm^−1^ in the spectrum of the GMO/C-I complex. This signal can be reasonably ascribed to the antisymmetric vibrations associated with the C=C of the phenyl ring of C-I since the modes localized on this portion of the molecule are strongly enhanced by the resonance effect of the 228 nm excitation wavelength. Moreover, these finding supports the hypothesis that the phenyl moieties interact with the lipid phase in the complex.

Figure 4e–h show a comparison between the UVRR spectra of C-II before and after its loading in the three matrices of GMO, PHYT and DOPE.

Similarly to what observed for C-I, the loading of C-II gold compound in all the three matrices induces a broadening of the peak at ≈1590 cm^−1^ associated with the phenyl rings deformation modes. This result confirms the hypothesis of an interaction of the phenyl rings of the compounds with the lipid layer, as suggested by SAXS data. Additionally, an intense signal centered at ≈1700 cm^−1^ is found in the spectra of the complex PHYT/C-II, which can be reasonably assigned to a combination of different vibrational modes. This could suggest an additional interaction established between the phenyl rings of this compound and the PHYT matrix. Interestingly, the rising of the Raman signal at ≈1380 cm^−1^ already found in the spectrum of the complex GMO/C-I was observed also in the spectra of C-II loaded both in GMO and DOPE.

### 3.3. ATR-FTIR

**Figure 5:**
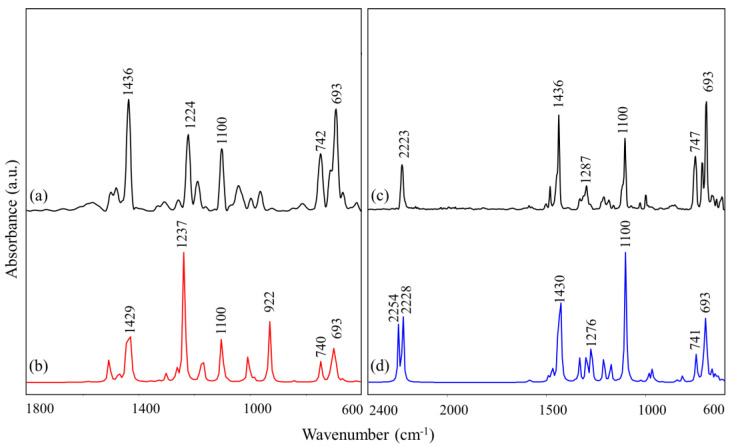
IR spectra of C-I (collected (**a**) and computed (**b**); 1800–600 cm^−1^ spectral range), and C-II (collected (**c**) and computed (**d**); 2400–600 cm^−1^ spectral range) drugs. The position (in terms of wavenumbers, cm^−1^) of the most significant peaks observed in C-I and C-II is shown.

The experimental ATR-FTIR spectra of C-I and C-II compounds acquired on crystalline samples and the corresponding theoretical ones are reported in Figure 5. Due to the presence of distinct substituents on the imidazole ring (Cl in C-I and C≡N in C-II) with different vibrational modes, spectra of C-I and C-II are shown in the 1800–600 cm^−1^ and 2400–600 cm^−1^ spectral ranges, respectively. A good correlation was found between the collected spectra and the computed ones, as shown in Figure 5. In particular, in both drugs, the following bands were detected: ≈1436 cm^−1^ (ν_asym_ C2–N and δC2–H); ≈1100 cm^−1^ (ν_asym_ C–P), and ≈747 cm^−1^ and ≈693 cm^−1^ (out-of-plane phenyl ring deformations). In addition, in C-I a band centered at 1224 cm^−1^ (symmetric in-plane imidazole ring deformation) was found, while in C-II, the bands at 1287 cm^−1^ (asymmetric in-plane imidazole ring deformation) and 2223 cm^−1^ (νC≡N) were observed.

**Figure 6:**
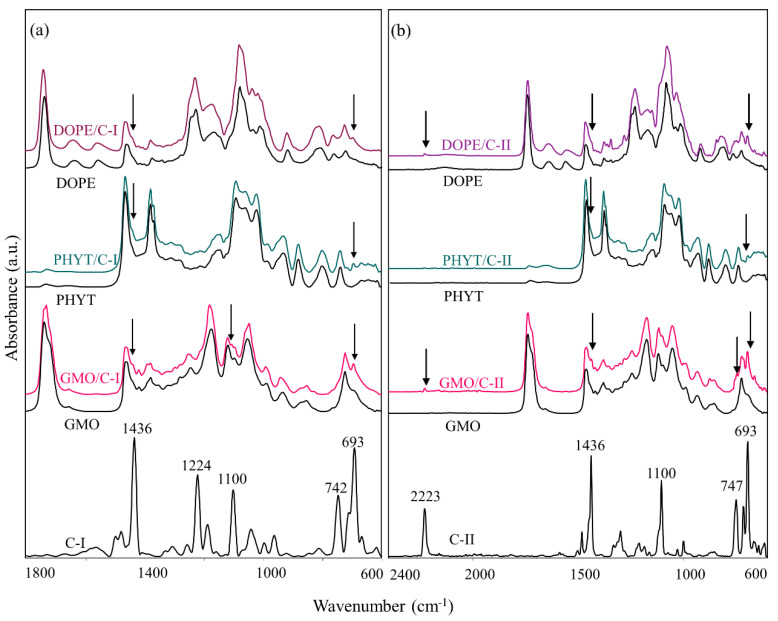
IR spectra of: (**a**) C-I pristine and loaded in the complexes C-I/PHYT, C-I/GMO, and C-I/DOPE (1800–900 cm^−1^ spectral range); (**b**) C-II pristine and loaded in the complexes C-II/PHYT, C-II/GMO, and C-II/DOPE (2400–900 cm^−1^ spectral range). The IR spectra of the lipid matrices PHYT, GMO, and DOPE are also reported for clarity. The position (in terms of wavenumbers, cm^−1^) of the most significant peaks observed in C-I and C-II is shown.

The ATR-FTIR spectra of pristine C-I and C-II compounds, together with those of the corresponding complexes with GMO, PHYT, and DOPE are shown in Figure 6a,b, respectively. For the sake of comparison, the IR spectra of the lipid commercial matrices GMO, PHYT, and DOPE are also shown [14,39,40,41]. As expected, the spectra of all the complexes are mainly constituted by the bands due to the vibrational modes of the lipid matrices. Nevertheless, it is possible to observe the rising of some signals clearly attributable to C-I and C-II compounds (evidenced by the black arrows in Figure 6a,b). In particular, in all C-I complexes, a shoulder at 1436 cm^−1^ and a small peak at 693 cm^−1^ (linked respectively to imidazole and phenyl ring vibrational modes) are detected. In the C-II complexes, besides these IR signals, a small peak at 2223 cm^−1^ is also observed (due to the nitrile groups on C4 and C5 of the imidazole ring). These findings confirm that the gold(I) compounds are encapsulated in the lipid matrices where they likely experience dispersion forces.

## 4. Conclusions

In this work, a novel study on the encapsulation of potential anticancer gold(I) compounds in nonlamellar lyotropic liquid crystalline lipid matrices has been presented. The loading of the compounds in three lipid systems was demonstrated using a combination of synchrotron radiation characterization techniques, namely SAXS, UV resonant Raman and ATR IR spectroscopy. This combined approach evidences the successful encapsulation of gold(I) compounds. In the ATR IR spectra of the loaded systems, the appearance of some typical vibrations of the gold compounds was observed. However, it was with SAXS and UVRR measurements that encapsulation of the gold(I) compounds in the lipid matrices, and particularly in the hydrophobic portion, was clearly highlighted. Gold(I) compound-induced phase transitions and/or changes in the lattice parameters of the host mesophases were evident from SAXS, whereas UV resonant Raman spectroscopy allowed us to focus on the specific spectral changes upon high-energy selective excitation of the gold molecules or part of them. Specifically, upon irradiation at 228 nm, we targeted the drugs’ phenyl moieties, revealing the modification of their typical vibrations caused by dispersion interactions with the lipid chains. Overall, these results demonstrate that the herein used lipid matrices can be considered promising candidates for the encapsulation of metal-based drugs. Moreover, the combination of the different techniques applied in this work represents a suitable method for the characterization of systems where cooperative dispersion forces occur.

## Figures and Tables

**Table 1:** Phase structure, lattice parameters and diameter of aqueous channels of GMO, PHYT and DOPE empty and loaded.

Sample	Phase	Lattice Parameter (nm)	Average Diameter of the Core Channel (nm)
GMO	*Im3m*	13.24	4.49
GMO/C-I	*H_II_*	6.09	2.49
GMO/C-II	*H_II_*	6.13	2.53
PHYT	*Pn3m*	6.54	2.31
PHYT/C-I	*H_II_*	4.90	2.10
PHYT/C-II	*H_II_*	5.01	2.21
DOPE	*H_II_*	7.31	2.90
DOPE/C-I	*H_II_*	7.03	2.62
DOPE/C-II	*H_II_*	7.25	2.84

## Supplementary Materials

The following are available online at https://www.mdpi.com/2079-4991/10/9/1851/s1, Figure S1: ^1^H NMR of GMO/F127 in CDCl_3_, Figure S2: ^1^H NMR of C-I in CDCl_3_, Figure S3: ^1^H NMR of C-II in CDCl_3_, Figure S4: ^1^H NMR of GMO/F127/C-I in CDCl_3_, Figure S5: ^1^H NMR of GMO/F127/C-II in CDCl_3_, Figure S6: ^1^H NMR of GMO/F127/C-I in D_2_O, Figure S7: ^1^H NMR of GMO/F127/C-II in D_2_O, Figure S8: ^31^P NMR C-I in CDCl_3_, Figure S9: ^31^P NMR C-II in CDCl_3_, Figure S10: ^31^P NMR of GMO/F127/C-I in CDCl_3_, Figure S11: ^31^P NMR of GMO/F127/C-II in CDCl_3_, Figure S12: ^31^P NMR of GMO/F127/C-I in D_2_O, Figure S13: ^31^P NMR of GMO/F127/C-II in D_2_O.
