# Supplementary material for: Synchrotron Characterization of Hexagonal and Cubic Lipidic Phases Loaded with Azolate/Phosphane Gold(I) Compounds: A New Approach to the Uploading of Gold(I)-Based Drugs
Source: Nanomaterials (Basel). 2020 Sep 16;10(9):1851. doi: 10.3390/nano10091851 (PMC7558674; doi:10.3390/nano10091851)
Supplement: Supplementary file 1 [file nanomaterials-10-01851-s001.pdf]

Supplementary Materials

Synchrotron Characterization of Hexagonal and Cubic Lipidic Phases Loaded with Azolate/Phosphane Gold(I) Compounds: A New Approach to the Uploading of Gold(I)-Based Drugs

Paola Astolfi [1], Michela Pisani [1,*], Elisabetta Giorgini [2], Barbara Rossi [3], Alessandro Damin [4], Francesco Vita [1], Oriano Francescangeli [1], Lorenzo Luciani [5] and Rossana Galassi [5]

[1] Dipartimento SIMAU, Università Politecnica delle Marche, Via Brecce Bianche, I-60131 Ancona, Italy; p.astolfi@univpm.it (P.A.); f.vita@univpm.it (F.V.); o.francescangeli@univpm.it (O.F.)

[2] Dipartimento DiSVA, Università Politecnica delle Marche, Via Brecce Bianche, I-60131 Ancona, Italy; e.giorgini@univpm.it

[3] Elettra-Sincrotrone Trieste S.C.p.A., S.S. 14-km 163.5, Basovizza, I-34149 Trieste, Italy; barbara.rossi@elettra.eu

[4] Department of Chemistry, NIS Centre and INSTM Reference Centre University of Turin, Via G. Quarello 15, I-10135 Turin, Italy; alessandro.damin@unito.it

[5] Scuola di Scienze e Tecnologie, Divisione Chimica, Università di Camerino, I-62032 Via Sant'Agostino 1, Italy; rossana.galassi@unicam.it (R.G.); lorenzo.luciani@unicam.it (L.L.)

* Correspondence: m.pisani@univpm.it; Tel.: +39-0712204263

NMR spectra

^{1}H and ^{31}P NMR spectra on GMO/F127/C-I and C-II samples were recorded after addition of water to GMO/F127/gold compound chloroform solutions taken to dryness and sonication. C-I and C-II were added at 10% w/w with respect to GMO.

^{1}H NMR spectra of GMO/F127, C-I and C-II in $CDCl_3$ are shown in Figures S1, S2 and S3 whereas those of the corresponding complexes are shown in Figures S4 and S5. Addition of D_2O induces the broadening of the signals Figure S6 and S7.

^{31}P NMR spectra of C-I and C-II in $CDCl_3$ are reported in Figures S8 and S9 and no variation in the chemical shift is observed when dissolved in $CDCl_3$ also in the presence of GMO/F127 (Figures S10 and S11).

^{31}P NMR signal of GMO/F127/C-I and C-II (Figures S12 and S13) disappears when the spectra are recording in D_2O likely because C-I and C-II compounds are not in solution but entrapped within the dispersed hexagonal phase.

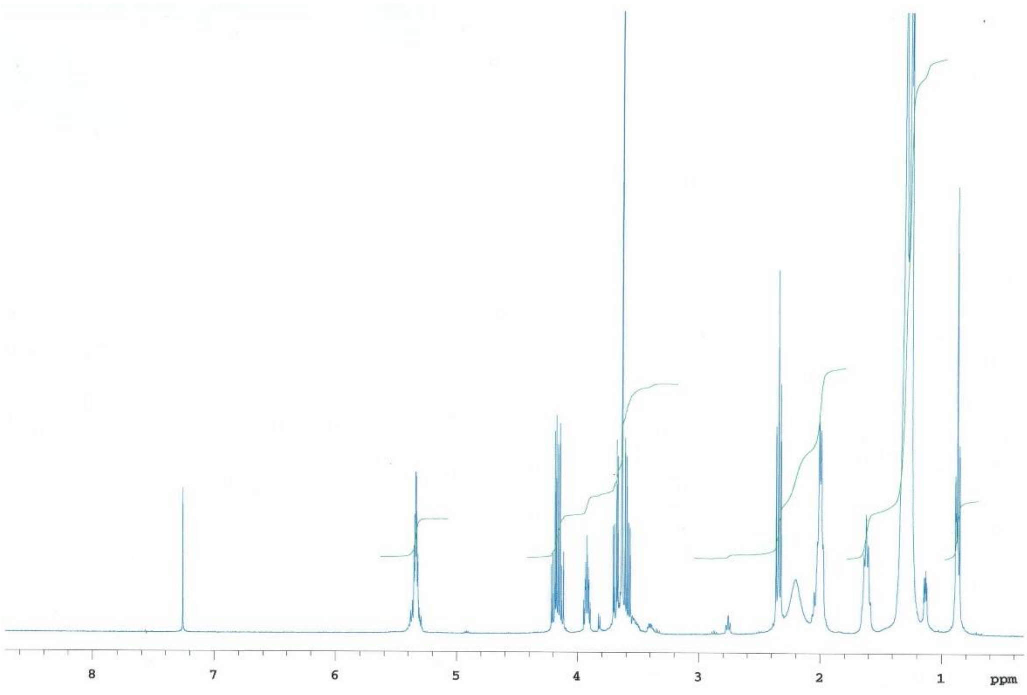

Figure S1. ^{1}H NMR of GMO/F127 in $CDCl_3$.

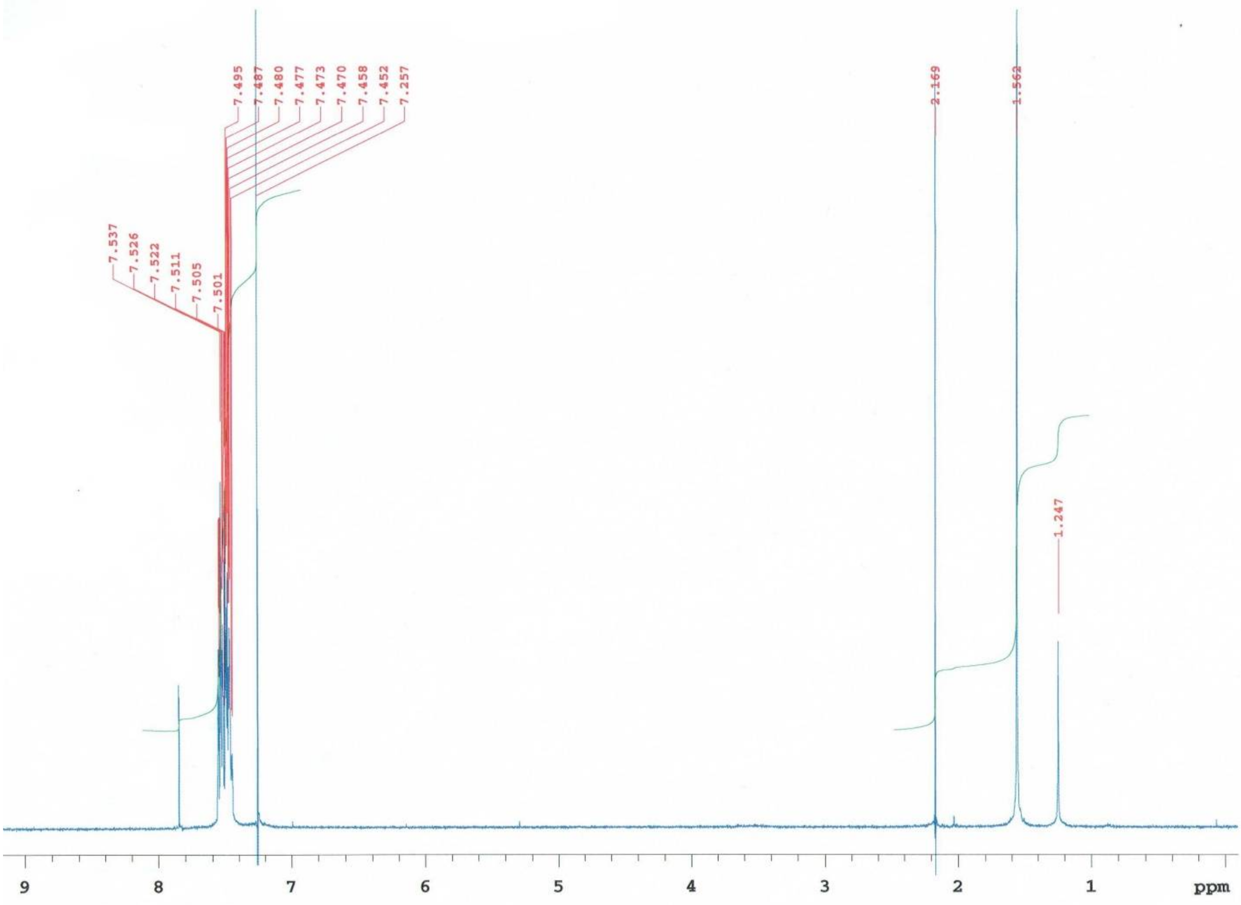

Figure S2. ^{1}H NMR of C-I in $CDCl_3$.

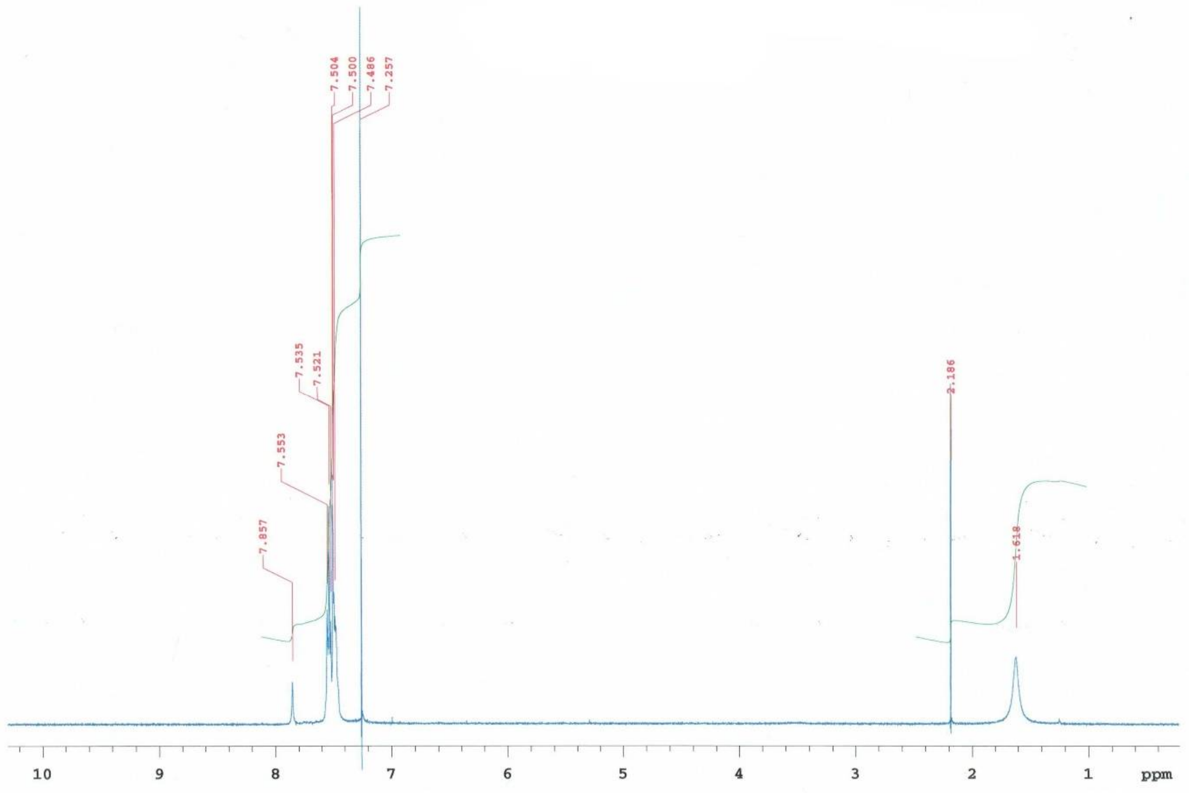

Figure S3. ^{1}H NMR of C-II in $CDCl_3$.

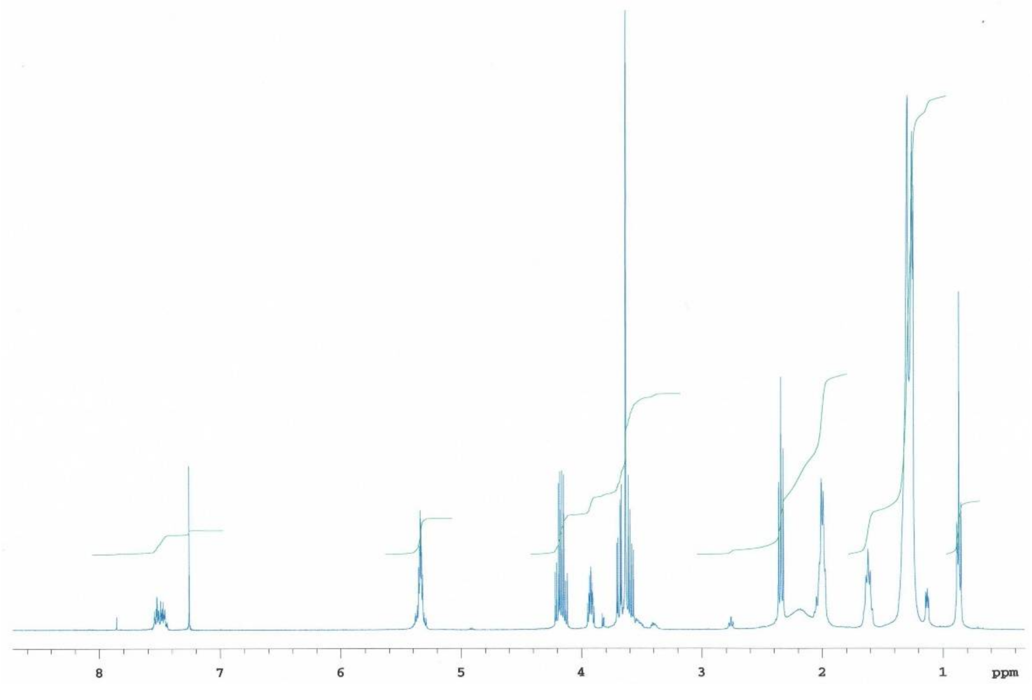

Figure S4. ^{1}H NMR of GMO/F127/C-I in $CDCl_3$

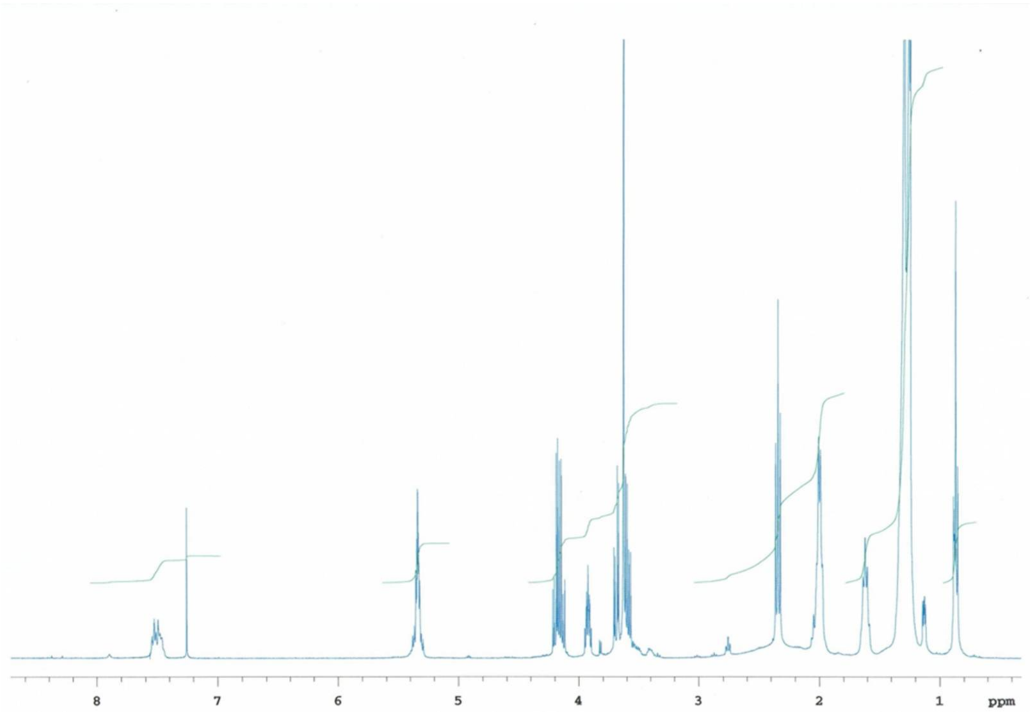

Figure S5. ^{1}H NMR of GMO/F127/C-II in $CDCl_3$

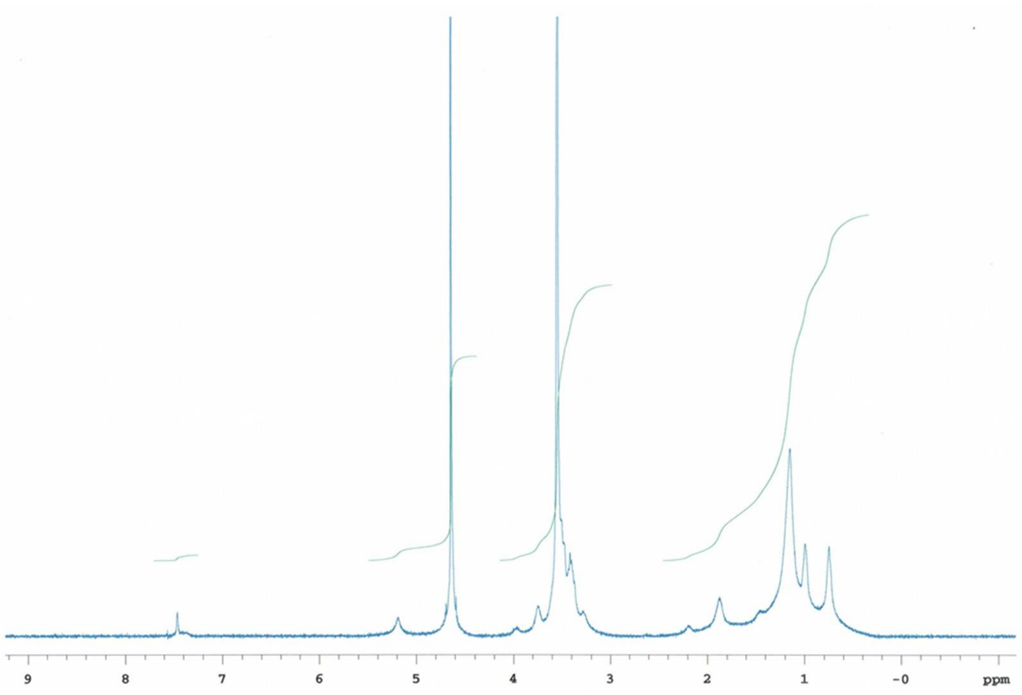

Figure S6. ^{1}H NMR of GMO/F127/C-I in D_2O

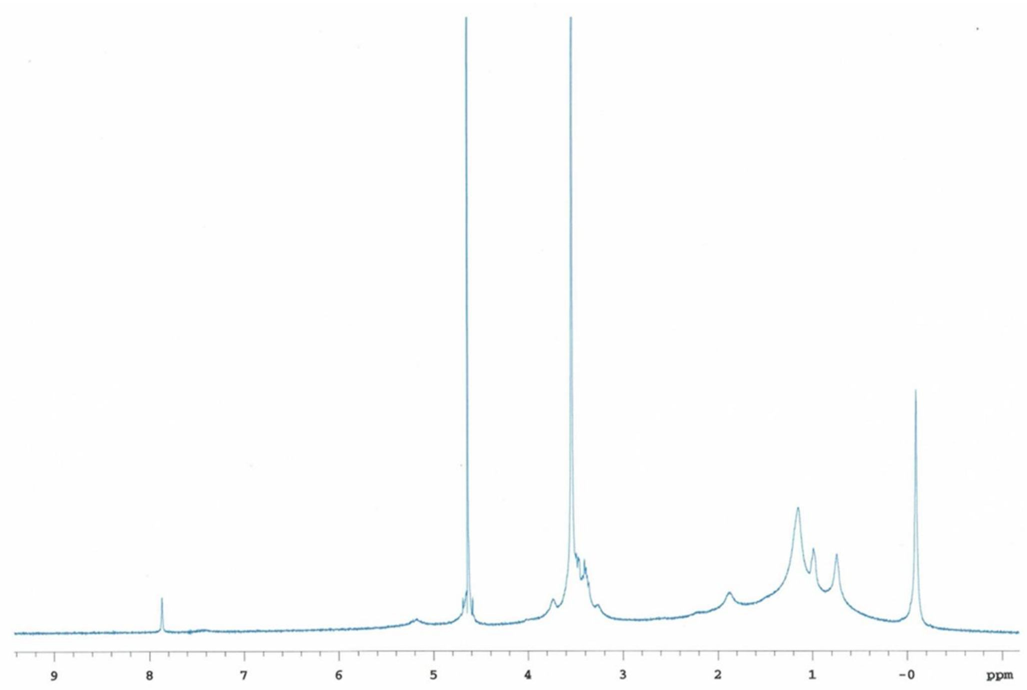

Figure S7. ^1H NMR of GMO/F127/C-II in D_2O.

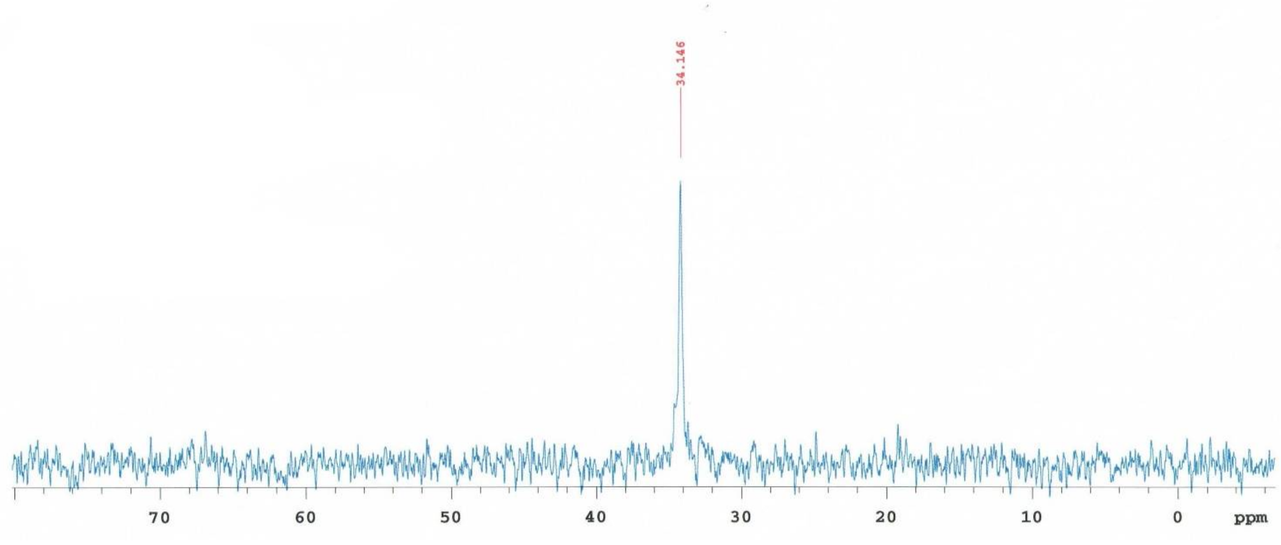

Figure S8. ^{31}P NMR of C-I in $CDCl_3$.

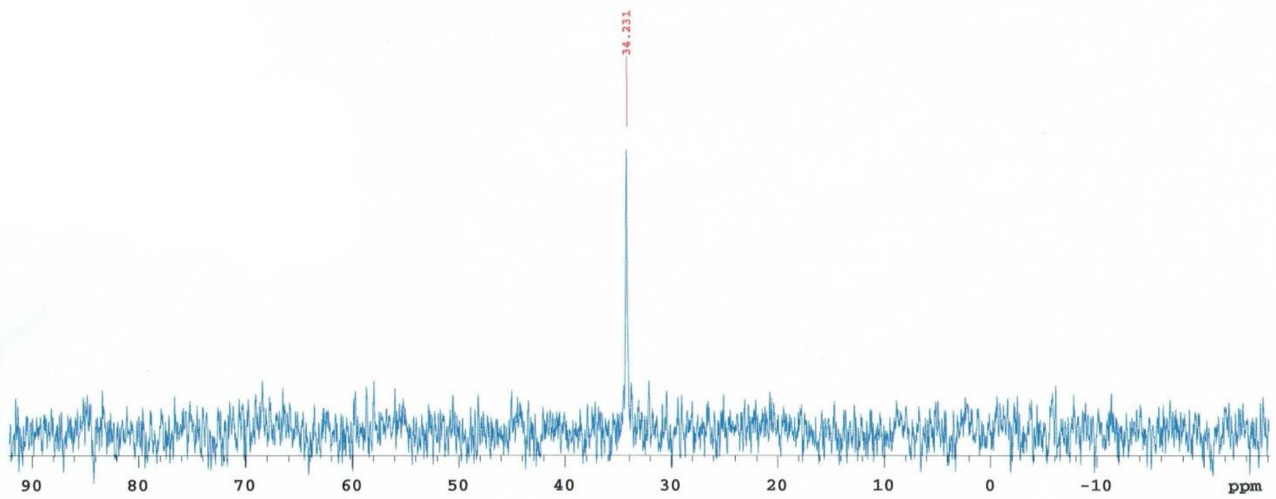

Figure S9. ^{31}P NMR of C-II in $CDCl_3$.

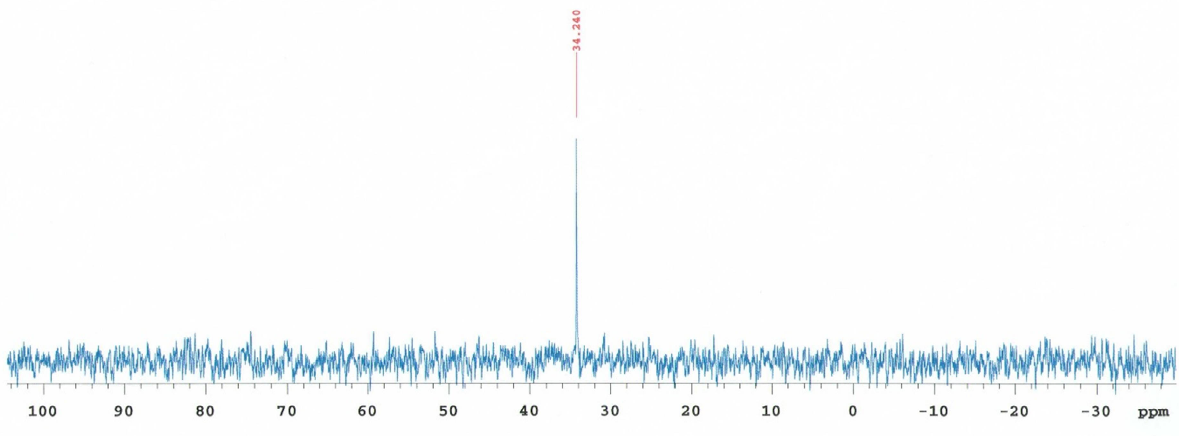

Figure S10. ^{31}P NMR of GMO/F127/C-I in $CDCl_3$

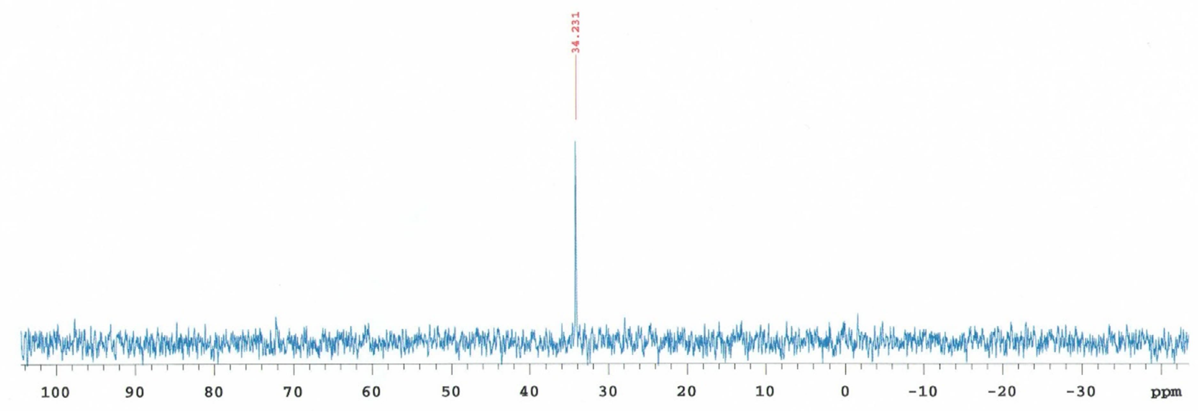

Figure S11. ^{31}P NMR of GMO/F127/C-II in $CDCl_3$

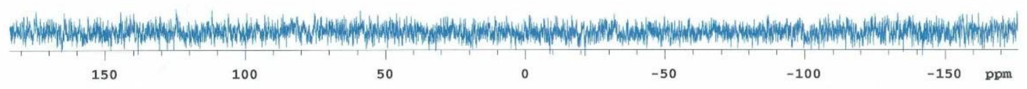

Figure S12. ^{31}P NMR of GMO/F127/C-I in D_2O

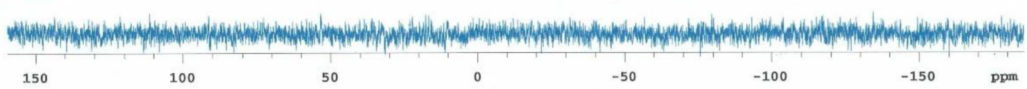

Figure S13. ^{31}P NMR of GMO/F127/C-II in D_2O
